# Application of fluorocarbon nanoparticles of ^131^I-fulvestrant as a targeted radiation drug for endocrine therapy on human breast cancer

**DOI:** 10.1186/s12951-024-02309-7

**Published:** 2024-03-12

**Authors:** Li Zhi, Chen Cheng, Luo Jing, Peng Zhi-Ping, Yang Lu, Tian Yan, Wang Zhi-Gang, Yin Guo-Bing

**Affiliations:** 1 Department of Breast and Thyroid Surgery, the Second Affiliated Hospital of Chongqing Medical University, 74 Linjiang Road, Yuzhong District, Chongqing, 400010 People’s Republic of China; 2 Department of Pathology, the First Affiliated Hospital of Chongqing Medical University, Chongqing, 400010 People’s Republic of China; 3 Department of Nuclear Medicine Laboratory, Chongqing Medical University, Chongqing, 400010 People’s Republic of China; 4 Department of Nuclear Medicine, the Second Affiliated Hospital of Chongqing Medical University, Chongqing, 400010 People’s Republic of China; 5 Department of Ultrasound Research Institute, the Second Affiliated Hospital of Chongqing Medical University, Chongqing, 400010 People’s Republic of China

**Keywords:** Breast cancer, Nanomedicine, Fulvestrant, Cerenkov radiation, Nuclear medicine, Photodynamic therapy

## Abstract

**Background:**

Breast cancer is the most prevalent malignant tumor among women, with hormone receptor-positive cases constituting 70%. Fulvestrant, an antagonist for these receptors, is utilized for advanced metastatic hormone receptor-positive breast cancer. Yet, its inhibitory effect on tumor cells is not strong, and it lacks direct cytotoxicity. Consequently, there's a significant challenge in preventing recurrence and metastasis once cancer cells develop resistance to fulvestrant.

**Method:**

To address these challenges, we engineered tumor-targeting nanoparticles termed ^131^I-fulvestrant-ALA-PFP-FA-NPs. This involved labeling fulvestrant with ^131^I to create ^131^I-fulvestrant. Subsequently, we incorporated the ^131^I-fulvestrant and 5-aminolevulinic acid (ALA) into fluorocarbon nanoparticles with folate as the targeting agent. This design facilitates a tri-modal therapeutic approach—endocrine therapy, radiotherapy, and PDT for estrogen receptor-positive breast cancer.

**Results:**

Our in vivo and in vitro tests showed that the drug-laden nanoparticles effectively zeroed in on tumors. This targeting efficiency was corroborated using SPECT-CT imaging, confocal microscopy, and small animal fluorescence imaging. The ^131^I-fulvestrant-ALA-PFP-FA-NPs maintained stability and showcased potent antitumor capabilities due to the synergism of endocrine therapy, radiotherapy, and CR-PDT. Throughout the treatment duration, we detected no notable irregularities in hematological, biochemical, or histological evaluations.

**Conclusion:**

We've pioneered a nanoparticle system loaded with radioactive isotope ^131^I, endocrine therapeutic agents, and a photosensitizer precursor. This system offers a combined modality of radiotherapy, endocrine treatment, and PDT for breast cancer.

**Graphical Abstract:**

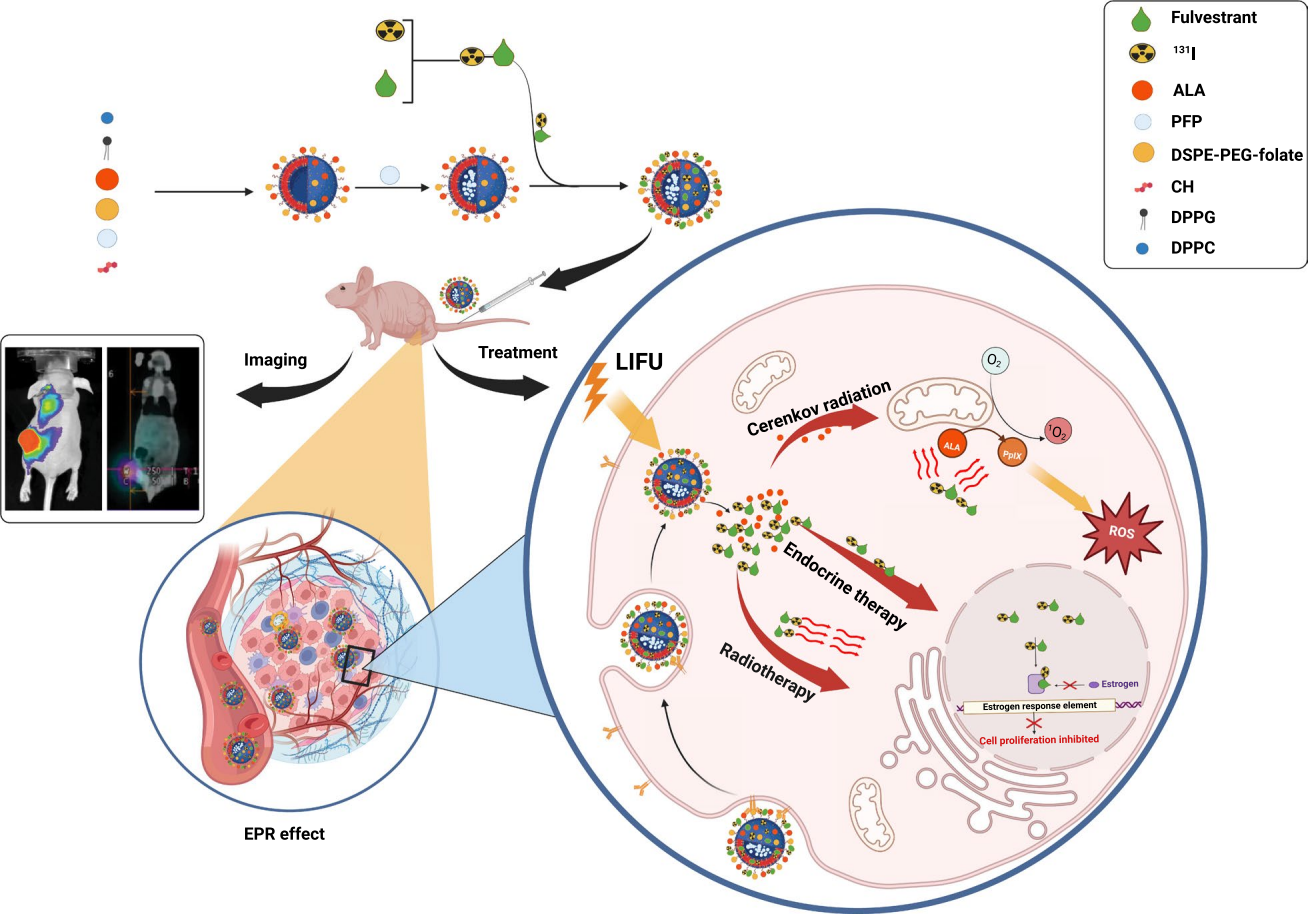

## Introduction

Breast cancer is the most prevalent malignant tumor in women [1], with its classification rooted in the expression levels of key molecular biomarkers: estrogen receptors, progesterone receptors, and human epidermal growth factor receptor 2 (HER2) [2]. Among these, hormone receptor-positive breast cancer emerges as the dominant type, constituting 70% of all cases. Specifically for estrogen receptor-positive breast cancer, endocrine therapy remains a cornerstone treatment [3, 4]. Fulvestrant, a cutting-edge steroidal estrogen receptor antagonist, is prescribed for postmenopausal women battling advanced metastatic breast cancer. It's particularly employed when initial endocrine therapy ceases to be effective [5]. Notably, fulvestrant's primary role is to suppress tumor cell proliferation without directly inducing cytotoxicity. This creates a conundrum: resistance to fulvestrant can lead to potential recurrence and metastasis. Hence, innovating new therapeutic strategies to amplify its effects or counteract drug resistance becomes paramount [6].

Targeted radionuclide therapy (TRNT) is emerging as a promising avenue in contemporary cancer treatment [7]. In TRNT, therapeutic radionuclides home into specific lesions [8], either by natural physiological uptake or by binding to specialized carriers like antibodies, peptides, and certain small molecules. These carriers are designed with a keen specificity and affinity for tumor cells. As a result, TRNT delivers precise radiation to tumor sites, sparing adjacent healthy tissues. This specificity becomes especially advantageous when treating systemic malignant tumors that might exhibit adverse reactions to external radiation therapies, such as metastatic ones. Historically, several radionuclides, including ^177^Lu, ^131^I, and ^111^In, have been harnessed to craft TRNT drugs for breast cancer [9]. In our preliminary research, we highlighted the therapeutic prowess of ^131^I-fulvestrant against hormone receptor-positive breast cancer. By labeling the fulvestrant with ^131^I, we transformed it into a radioactive ^131^I carrier. Given fulvestrant's intrinsic binding capability with estrogen receptors, this radioactive ^131^I-fulvestrant zeroes in on estrogen receptor-positive breast cancer cells. This approach orchestrates a dual therapeutic synergy, fusing radiation and endocrine therapy [10]. Estrogen receptors, while found in tumor cells, are also ubiquitously expressed across various organs in the human body. This widespread expression and lack of specificity compromise the therapeutic efficiency of ^131^I-fulvestrant. Moreover, the intricate tumor microenvironment poses a hurdle as drugs must navigate numerous physiological barriers to reach tumor tissues. Fluorocarbon nanoparticles present a targeted drug delivery system that addresses these challenges [11].

Folate (FA) is recognized as one of the most effective targeting agents for solid tumor cells due to the overexpression of folate receptors in a range of solid tumors, including ovarian, lung, and breast cancers [12, 13]. Upon binding with its receptor, folate initiates cellular internalization of the receptor-FA complex and its attached cargo through endocytosis, making this receptor an attractive target for anticancer drugs. Numerous studies have employed FA to decorate their nanostructures, aiming for targeted cancer cell delivery and tumor-specific drug release in various cancer models [14–16]. Specifically, folate-decorated bovine serum albumin-stabilized gold nanoparticles (BSA-GNPs) have demonstrated significant advantages in enhancing the efficacy of radiotherapy for breast cancer, coupled with commendable biocompatibility [17]. Beyond strategies involving BSA-GNPs nanoparticles, in the treatment of glioma, folate and BSA-modified gold nanoclusters (FA-AuNCs) have shown remarkable effects in extending the survival time of rats bearing gliomas, owing to their small size and specific targeting capabilities [18]. Numerous similar studies underscore folate as a prime targeting agent for tumor cells, reaffirming its role in advancing the precision of cancer treatments [19–24].

We integrated ^131^I-fulvestrant with folate-linked fluorocarbon phase-change nanoparticles to craft a radioactive ^131^I-fulvestrant fluorocarbon nanoparticle compound. Leveraging the enhanced permeability and retention (EPR) effect, this nanoparticle compound not only navigates through the endothelial gaps in cancerous tissue capillaries with ease but also amasses at tumor sites [25], specifically binding to breast cancer cells that have an abundance of folate receptors. Furthermore, perfluoropentane (PFP), a fluorinated aliphatic compound, stands out not only as an efficient drug carrier but also as a binder to potent tumor-targeting molecules, which amplifies drug accumulation within tumor tissues. Upon exposure to ultrasonic irradiation, these nanoparticles undergo a phase transition, rupturing to release the contained radioactive ^131^I-fulvestrant that then specifically binds to estrogen receptors. Concurrently, the explosive effect from the nanoparticle's liquid-to-gas phase transition amplifies tumor cell permeability, thereby boosting the intracellular concentration of the drug [26].

Combination therapies, in contrast to single-agent treatments, provide the advantage of synergism. This collaboration amplifies the potential to combat multidrug resistance and enhances antitumor efficacy. Photodynamic therapy (PDT) stands out in this spectrum. It's a targeted therapy, dependent on external stimuli, that employs photosensitizers (PSs). Upon exposure to specific wavelengths of light, these PSs generate reactive oxygen species (ROS), leading to cancer cell death. PDT, being clinically endorsed and with its low systemic toxicity and non-invasive approach, is increasingly gaining traction [27]. However, a significant limitation exists: the tissue penetration capacity of the light required for PDT is minimal, rendering it inefficient against deeply embedded tumors [28]. PDT induced by Cerenkov radiation (CR-PDT) is a beacon of hope for this challenge. Cerenkov radiation arises during the decay of radioactive nuclides. Fundamentally, when charged particles (e.g., positrons) traverse a medium faster than light's speed within that medium, they excite the medium. This excited state soon reverts to its ground state, releasing electromagnetic radiation [29]. Using this principle, CR-PDT exploits the Cerenkov radiation from adjacent radioactive nuclides to activate PSs. This process yields detrimental ROS, positioning CR-PDT as a promising remedy for the depth challenges faced by traditional PDT [30]. Nevertheless, an imminent issue is preventing the photosensitizer from prematurely reacting with Cerenkov radiation during the drug-making process. Addressing this, we turned to the tumor's rich mitochondrial profile, from which we crafted a molecular switch. ALA serves as the natural precursor to protoporphyrin IX (PpIX), a widely used photosensitizer precursor in clinical PDT. Notably, the transformation of ALA into PpIX is mitochondria-intensive [31]. Capitalizing on the abundant mitochondrial content within tumor cells, ALA undergoes conversion into PpIX specifically at tumor sites. Once formed, PpIX is activated by the Cerenkov radiation (CR) emanating from the decay of ^131^I, leading to the production of ROS that destroys the tumor cells. Conversely, in regular tissues with a lesser mitochondrial density, the ALA-to-PpIX conversion remains minimal, ensuring limited damage. It's also worth highlighting that the dominant emissions of CR are situated within the ultraviolet and blue regions of the spectrum. This spectral alignment makes CR particularly apt for energizing photosensitizers like PpIX, which are sensitive to ultraviolet and blue wavelengths [32].

Building on the conceptual foundation, we conceptualized a novel iodinated fulvestrant nanoparticle termed ^131^I-fulvestrant-ALA-PFP-FA-NPs. In our first step, we labeled fulvestrant with radioactive ^131^I, transforming it into a carrier for this radioactive isotope, resulting in ^131^I-fulvestrant. Subsequently, this was amalgamated with fluorocarbon phase-change nanoparticles infused with folic acid and ALA, yielding a composite nanoparticle enriched with radioactive ^131^I-fulvestrant. The prime targeting mechanism for this composite nanoparticle is via folic acid receptors, steering the drug-laden nanoparticles directly to breast cancer lesions. When exposed to low-intensity focused US (LIFU), a phase transition is triggered in the tumor vicinity. This transition facilitates tumor cell ablation and boosts cellular permeability. Following this, the ^131^I-fulvestrant is released. With its intrinsic affinity for estrogen receptors, it selectively binds to breast cancer cells. Parallel to this, the profuse mitochondria in the tumor sites facilitate the transformation of ALA into the active photosensitizer, PpIX. Upon being excited by CR, this photosensitizer spawns ROS, further compromising the tumor cells. Such a strategic alignment promises a pioneering multi-modal treatment avenue for breast cancer, incorporating targeted radiotherapy, photodynamic therapy, and collaborative endocrine therapy.

## Material and methods

### Material

Fulvestrant, purchased from Hangzhou Rongda Pharmaceutical Co., Ltd.; Chloramine T, purchased from Chengdu Kelong Chemical Reagent Factory; Sodium metabisulfite, purchased from Chengdu Kelong Chemical Reagent Factory; Na 131I solution was supplied by Chengdu CNNC Gaotong Isotope Co., Ltd., with a radioactivity of about 5mci/ml. The real-time radioactivity must be calculated when used. MCF-7 cells were sourced from the Cell Bank of the Chinese Academy of Sciences Committee for Type Culture Collection; Female Balb/c nude mice (21 days old) were from Chongqing Medical University Experimental Animal Center. PFP was purchased from Sigma-Aldrich (St. Louis, MO, USA). Dipalmitoylphosphatidylcholine (DPPC), 1,2-Distearoyl-sn-glycero-3-phosphoethanolamine-N-[methoxy(poly(ethylene glycol))- 2000] (DSPE-PEG2000), and cholesterol were obtained from Avanti Polar Lipids, Inc. (Alabaster, AL, USA). 1,1'-Dioctadecyl-3,3,3',3'-Tetramethylindocarbocyanine Perchlorate (DiI) and 4′,6-diamidino-2-phenylindole (DAPI) were purchased from Beyotime Technology.

### Preparation of ^131^I-fulvestrant

To prepare the ^131^I-fulvestrant, the fulvestrant was radiolabeled with ^131^I using an optimized Chloramine T (Ch-T) method. Initially, 50 µl of fulvestrant was combined with 100 µl of Chloramine T in a standard EP tube. Subsequently, 1 mCi of Na^131^I was added, followed by vigorous shaking for uniform mixing. This mixture was then allowed to incubate at room temperature for 5 min. To cease the iodination reaction, 200 µl of sodium metabisulfite was introduced into the tube. The solution was incubated again at room temperature, ensuring a pH of 7.5, for 5 min. This termination step was performed five times.

### Determination of ^131^I-fulvestrant labeling efficiency

To determine the labeling efficiency of ^131^I-fulvestrant, paper chromatography was employed. A chromatography paper was sectioned into a 1 × 20 cm strip and folded lengthwise. Following centrifugation, the supernatant and the Na^131^I solution were aspirated using a capillary tube, retaining approximately 1 cm of the liquid at the bottom. Once dried, the paper's base was immersed roughly 0.5 cm deep in ethanol and shielded using a 2000 mL beaker. The paper was removed when the eluent's front edge reached a distance of 10 cm from the sample spot. Subsequent drying at 37 °C was followed by sectioning the paper into 0.5 cm strips, starting from the 1 cm segment at the bottom. Using a gamma counter, the radioactivity (cpm) of each strip was measured. The labeling efficiency was then calculated using the subsequent formula:

$$Labeling\,rate\, = \,\frac{{Radioactivity\,of\,the\,marker^{\prime}s\,paper}}{Radioactivity\,of\,all\,paper\,strips}\, \times \,100\%$$

### Preparation and characterization of ^131^I-fulvestrant-ALA-PFP-FA-NPs

A lipid film was prepared by combining 16 mg each of phosphatidylcholine (DPPC), folate-coupled phospholipid (DSPE-PEG-folate), glycerophospholipid (DPPG), and cholesterol (CH) in a molar ratio of 69:8:8:12 in a round-bottom flask with 10 mL of chloroform. After ensuring complete dissolution in a water bath, the chloroform was evaporated using a rotary evaporator set at 52 ℃ and 90 rpm for an hour. The resulting lipid film was rehydrated with 2 mL of PBS buffer, ultrasonicated to ensure detachment, and the suspension was set aside in a 10 mL EP tube. Separately, 5-Aminolevulinic acid (ALA) was mixed with ^131^I-fulvestrant in a 2 mL EP tube in a molar ratio of 4:1:1 relative to DPPC. This mixture was dissolved in 200 μL of deionized water, followed by the addition of 200 μL of PFP. The ALA and PFP were emulsified under ice bath conditions using a sonicator. Post-emulsification, this mixture was combined with the lipid suspension, subjected to a second round of sonication, and then centrifuged and washed thrice. The final liposomes, termed ^131^I-fulvestrant-ALA-PFP-FA-NPs, were resuspended in 1 mL of PBS buffer and stored at 4 ℃.

Transmission electron microscopy (TEM) was used to observe the morphology and other characteristics of drug-loaded targeted liquid fluorocarbon nanoparticles.

Transmission Electron Microscopy (TEM) was employed to observe the morphological and inherent characteristics of the drug-loaded targeted liquid fluorocarbon nanoparticles. Additionally, their particle size and Zeta potential were determined using a nanoparticle size analyzer, specifically a Malvern instrument. During the preparation process of the drug-loaded targeted nanoparticles, supernatants from each step were collected, and filtered, and the remaining drug concentrations were subsequently analyzed with High-Performance Liquid Chromatography (HPLC). The HPLC setup utilized a reverse-phase C-18 column (150mmx4.6 mm, 5 μm, C18) with a mobile phase consisting of acetonitrile: water in a 50:50 volume ratio. The flow rate was set at 1 ml/min, the injection volume was 20 μL, and the UV detection was done at a wavelength of 227 nm. Based on these readings, encapsulation efficiency and drug loading were then calculated using the subsequent formula:

$$Encapsulation\,rate\,\left( \% \right)\, = \,\frac{Nanoparticle\,Drug\,Content}{{Drug\,Administration\,Dosage}}\, \times \,100\%$$

$$\begin{aligned} & {\text{Drug}}\,{\text{Loading}}\,{\text{Capacity}}\,\left( {{\% }} \right)\, \\ &\quad= \,\frac{Nanoparticle\,Drug\,Content}{{Mass\,of\,Nanoparticles}}\, \times \,100{\text{\% }} \end{aligned}$$

### Detection of ^131^I-fulvestrant-ALA-PFP-FA-NPs drug release in vitro

The in vitro release of drug-loaded nanoparticles was assessed using the dialysis method. For this, two dialysis bags with a molecular weight cut-off of 3500 were prepared: one as the experimental group and the other as the control group. Into each bag, 4 ml of the drug-loaded targeted nanoparticle suspension was introduced. Both bags were then placed in separate 50 ml beakers, each containing 30 ml of PBS buffer solution. The experimental group underwent LIFU irradiation, while the control group remained untreated. Subsequently, both were kept on a thermostatic water bath shaker at 37℃, operating at a speed of 100r/min. For 14 days, 1 ml of solution was sampled from each beaker every 12 h for analysis. After each sampling, an equivalent volume of PBS was replenished. The samples' absorbance was analyzed using a UV spectrophotometer to derive the cumulative drug release curve, which provided insights into the drug release behavior, especially highlighting the role of ultrasound in promoting drug release.

### Ultrasound imaging in vitro

For the ultrasound imaging assessment, ^131^I-fulvestrant-ALA-PFP-FA-NPs were suspended in a PBS buffer solution and then embedded within a 4% agarose gel mold. Utilizing the Esaote MyLab90 ultrasound system (Florence, Italy), the imaging properties of ^131^I-fulvestrant-ALA-PFP-FA-NPs were captured over time under varying LIFU irradiation conditions. The echo signal intensity was specifically quantified within the designated region of interest (ROI).

### Cellular uptake assay of ^131^I-fulvestrant-ALA-PFP-FA-NPs

^131^I-fulvestrant-ALA-PFP-FA-NPs were labeled with Dil using the lipid insertion method, resulting in the formation of Dil-^131^I-fulvestrant-ALA-PFP-FA-NPs. MCF-7 cells were seeded onto confocal culture dishes. Once adhered to, varying concentrations of Dil-^131^I-fulvestrant-ALA-PFP-FA-NPs were introduced to the respective dishes and allowed to incubate for 6 h. Post-incubation, the culture medium was carefully discarded, and the cells were rinsed thrice with PBS. After each rinse, the PBS was carefully aspirated using a pipette tip. Cells were then fixed using paraformaldehyde and stained with DAPI for 15 min. After a thorough PBS wash to eliminate any residual DAPI, the dishes were sealed with an anti-fluorescence quenching agent. The cellular uptake, visualized by Dil fluorescence, was subsequently examined under a laser confocal microscope.

### Reactive Oxygen Species (ROS) content detection

MCF-7 cells were dispensed into a 12-well plate, with a seeding density of 2 × 10^5^ cells per well. Once the cells adhered firmly, they were treated with various drugs based on specific groupings and subsequently incubated for 4 h. Following this, the cells were rinsed thrice using sterile PBS. DCFH-DA was then introduced into the cell culture medium and the mixture was left to incubate for an additional 30 min. After incubation, the cells were harvested and their ROS content was assessed using a flow cytometer.

### Cell viability assay

To assess the cytotoxicity of drugs from the various groups, the CCK-8 assay was employed. MCF-7 cells were plated in a 96-well format, with each well containing a density of 6000 cells, and were allowed to culture for 24 h. Subsequently, nanoparticles representative of each group were introduced to the culture medium, followed by another 24-h incubation period. Post-incubation, cell viability was ascertained using the CCK-8 assay.

### Detection of Cerenkov radiation of ^131^I radioisotope

Cerenkov radiation from the 131I radioisotope was assessed under varied conditions using the IVIS system from PerkinElmer Inc. The test compounds, namely PBS, ^131^I + ALA, and ^131^I + PpIX, were all employed in their unbound forms. Image acquisition was conducted using diverse emission filters, ensuring that excitation was effectively blocked.

### Animal husbandry and handling

All animal procedures were conducted in accordance with the Guide for the Care and Use of Laboratory Animals and were approved by and conducted in accordance with the Institutional Animal Care and Use Committee of Chongqing Medical University (IACUC-CQMU-2023-0464). Female immunodeficient BALB/c nude mice (3 weeks) were bred and housed at temperatures between 19 °C and 22 °C with controlled humidity conditions. Food and water were provided ad libitum. The mice were acclimated for 7 days before the commencement of the study. To induce solid tumors, 1 × 10^7^ cells in 100 μL PBS (pH = 7.4) were subcutaneously injected into the left flank of each mouse. Tumor volumes and body weights were measured every other day, and tumor volumes were calculated using the specified formula. A standardized humane endpoint protocol was implemented for animal euthanasia. If, at any point during the research, signs of pain, significant tumor necrosis or infection [33], hemorrhaging, or extensive metastasis were observed, or if the tumor volume surpassed 1500 mm^3^, or there was a decrease in body weight exceeding 15%, the mice were humanely euthanized through intraperitoneal injection of pentobarbital sodium at 150 mg/kg. The tumor volumes were calculated according to the formula: $Tumor volume \left({mm}^{3} \right)=(Tumor length)\times {\left(Tumor width\right)}^{2}\times 0.5$

### SPECT/CT imaging and biodistribution study

To explore the biodistribution of ^131^I-fulvestrant-ALA-PFP-FA-NPs in MCF-7 tumor-bearing mice, we substituted ^131^I with ^125^I as a tracer. Utilizing the NM670 human SPECT/CT device by GE, USA, we captured SPECT/CT images to monitor the distribution of ^125^I-fulvestrant-ALA-PFP-FA-NPs in these mice. Both experimental and control mouse groups were administered with the nanoparticle solution (0.2 mCi, 0.2 ml), and images were subsequently obtained within a 48-h window post-administration.

### ^18^F-FDG Micro-PET/CT mouse imaging analysis

Mice underwent ^18^F-FDG Micro-PET/CT scans, utilizing the system from Siemens, Germany, stationed at Chongqing Medical University Nuclear Medicine Laboratory's Small Animal PET Research Center. These scans were executed pre-treatment and 24 h post-treatment. The ^18^F-FDG, procured from China National Nuclear Corporation's Isotope Co., Ltd., boasted a radiochemical purity exceeding 95%. Approximately 60 min before scanning, each mouse was administered a dose of 5.55 MBq (150 μCi) of ^18^F-FDG via tail vein injection. Pre-scan preparations also included the use of isoflurane. Image reconstructions were facilitated using the Inveon Acquisition Workplace software (Version 2.0 by Siemens preclinical solutions).

### In vivo fluorescence imaging

Mice-bearing tumors received an intravenous injection of DIR-labeled ^131^I-fulvestrant-ALA-PFP-FA-NPs through the tail vein. Post-injection, in vivo fluorescence imaging was carried out at various intervals. Following 48 h, the tumors and select tissues were surgically removed for further ex vivo imaging analyses.

### Animal therapeutic assessment

To evaluate the therapeutic efficacy of ^131^I-fulvestrant-ALA-PFP-FA-NPs, female Balb/c nude mice, 4 weeks of age, were subcutaneously inoculated with MCF-7 tumor cells. When the tumors grew to a volume of 90 mm^3^, the mice were categorized into six distinct groups, with five mice per group, for varied treatments. In these experiments, the administered dosages of ^131^I and ALA stood at 30 μCi/g and 30 μg/g, respectively. Mice in groups 2 and 3 received intramuscular injections in their right thigh muscles, while those in groups 1, 4, 5, and 6 were given the drugs through tail vein injections. Post-injection, precisely after 30 min, the tumor sites were exposed to LIFU irradiation (2.6 W/cm^2^ for 3 min), ensuring a 3 cm gap between the tumor and the LIFU instrument. Tumor dimensions were recorded every two day over 19 days. Survival outcomes were analyzed using the Kaplan–Meier approach, with endpoint criteria set at tumor size exceeding 1500 mm^3^, death, or weight loss surpassing 15%.

### In vivo toxicity assessment

To assess in vivo toxicity, groups were established and drugs were administered as previously mentioned. The overall health and well-being of the animals were monitored daily, and body weight measurements were taken every 3 days. Hematological and blood biochemical indicators, including aspartate aminotransferase (AST), alanine aminotransferase (ALT), blood urea nitrogen (BUN), creatinine (CRE), and alkaline phosphatase (ALP), were derived from whole blood samples using a biochemical analyzer. Furthermore, H&E staining was performed on blood and specific tissue samples, such as the heart, lungs, liver, kidneys, spleen, muscles, and thyroid.

### Histological analysis

For histological evaluation, organs and tumor tissues from the mice were procured. These samples were then fixed in 4% formaldehyde before being sectioned and stained using H&E, TUNEL, and Ki67.

### Statistical analysis

All experiments were performed in triplicate. Data are represented as mean ± standard deviation (SD). The analysis of variance (ANOVA) was conducted using SPSS 23.0 statistical software (SPSS Inc., Chicago, USA) to determine statistical differences between experimental groups. A P value of < 0.05 was deemed statistically significant.

## Results

### Synthesis and identification of ^131^I-fulvestrant

**Fig. 1:**
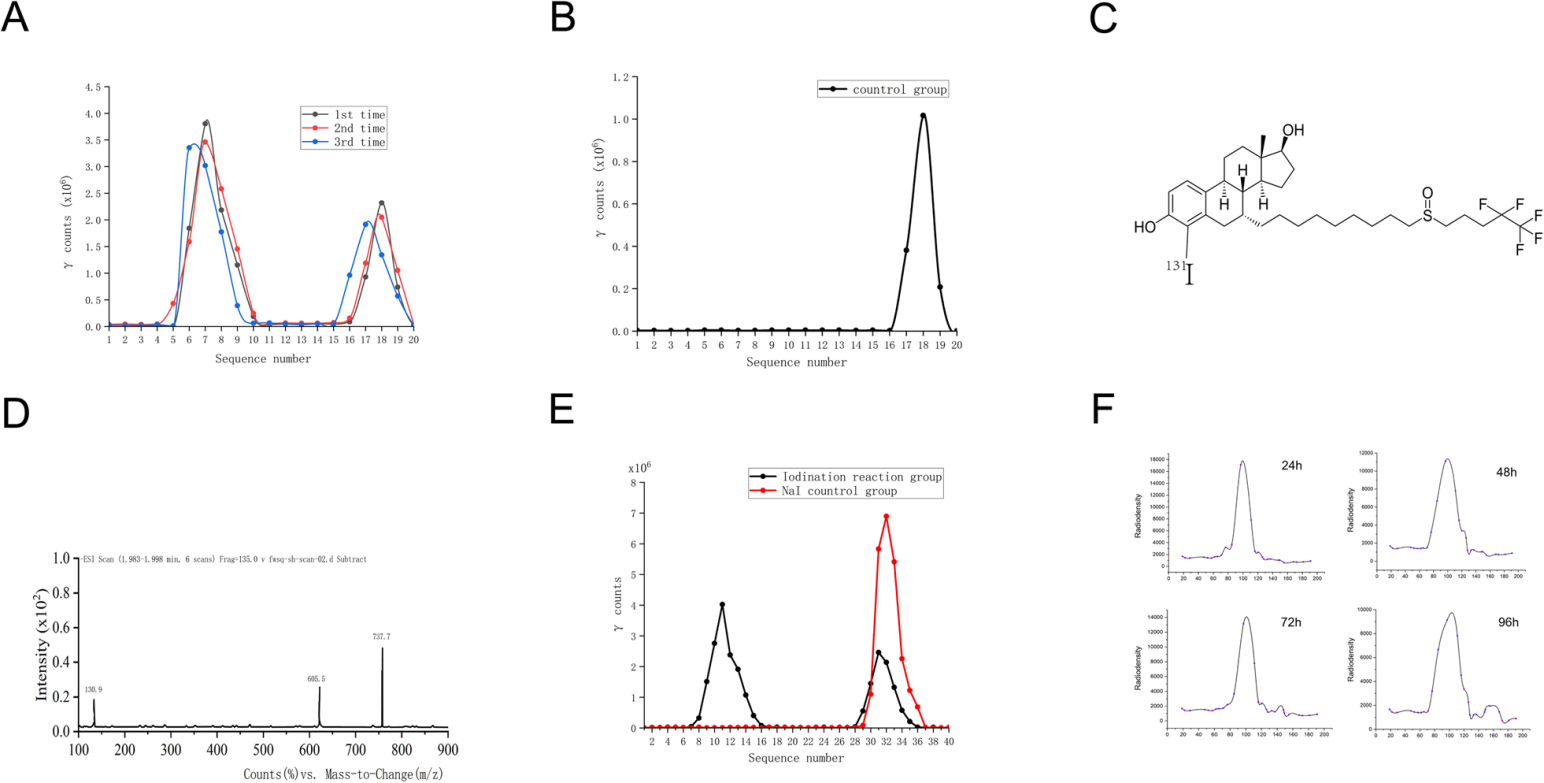
**Synthesis and identification of **^**131**^**I-fulvestrant: A** Results of paper chromatography for ^131^I-fulvestrant. **B** Results of paper chromatography for Na^131^I control group. **C** Chemical structure of ^131^I-fulvestrant. **D** Mass spectrometry identification results for ^131^I-fulvestrant. **E** Molecular sieve column chromatography purification results for ^131^I-fulvestrant. **F** Identification of the stability of ^131^I-fulvestrant at different time points using paper chromatography. (Values are means ± s.d., n = 3)

The synthesis of ^131^I-fulvestrant was achieved using the chloramine-T method. Its subsequent identification and separation were accomplished through paper chromatography, capitalizing on the differential diffusion rates of ^131^I-fulvestrant and free iodine. Figure 1A, B illustrates the successful synthesis of ^131^I-fulvestrant post-paper chromatography. Further validation of the synthesis was carried out using liquid chromatography-mass spectrometry, with the results presented in Fig. 1D. The purified ^131^I-fulvestrant, obtained using a chromatographic column, displayed a labeling rate of 70.45 ± 2.3% as depicted in Fig. 1E. The stability of the iodinated fulvestrant was also evaluated. Figure 1F reveals that paper chromatography and radioactivity measurements at 24 h, 48 h, and 72 h yielded a singular peak. However, a subtle second peak was discernible at 96 h. This secondary peak, found within the chromatographic band range of free iodine when compared to the control, suggests that the iodinated product remains stable for up to 96 h. The daily radioactivity decline aligns with the decay characteristics of ^131^I. The results indicate that ^131^I-fulvestrant has been successfully synthesized.

### Characterization of ^131^I-fulvestrant-ALA-PFP-FA-NPs

**Fig. 2:**
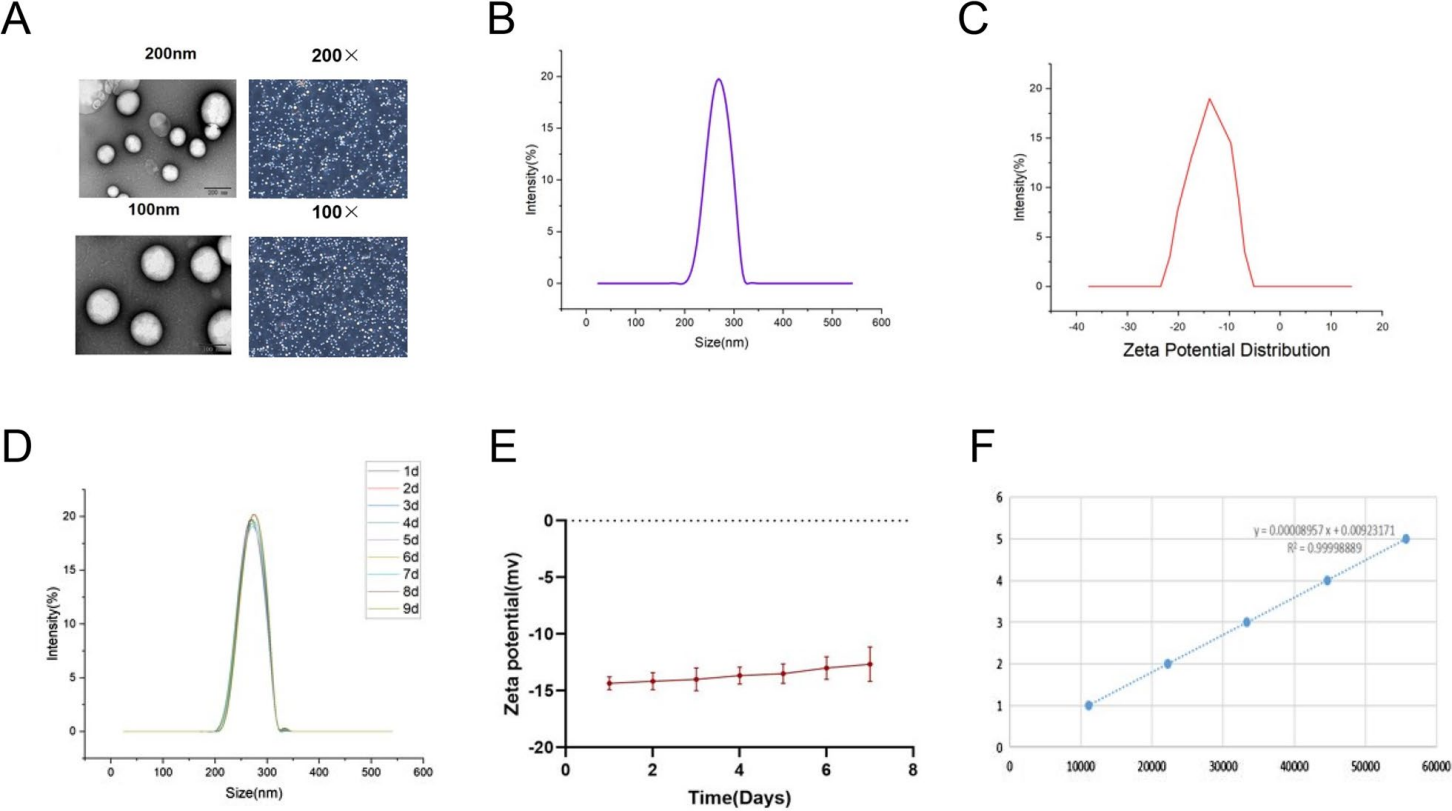
**Characteristics of **^**131**^**I-fulvestrant-ALA-PFP-FA-NPs: A** structural identification of ^131^I-fulvestrant-ALA-PFP-NPs using transmission electron microscopy and light microscopy. **B** Determination of nanoparticle size using nanoparticle size analyzer. **C** Measurement of nanoparticle zeta potential using malvern instrument. **D** Determination of nanoparticle stability (changes in particle size at different time points). **E** Changes in nanoparticle zeta potential at different time points. **F** Determination of nanoparticle encapsulation efficiency (assessment of nanoparticle radioactivity). (Values are means ± s.d., n = 3)

The synthesized ^131^I-fulvestrant-ALA-PFP-FA-NPs liposome appears as a white emulsion. Both transmission electron microscopy and optical microscopy results demonstrate that these liposomes are spherical, uniformly sized, and well-dispersed (Fig. 2A). Notably, the transmission electron microscopy images reveal a distinct shell-core spherical structure, with an average radius measuring 259 nm ± 5.8 nm (Fig. 2A). Validating this observation, the Malvern particle size analyzer recorded the diameter of the liposomes as 260 ± 6.4 nm (Fig. 2B). The Zeta potential of the nanoparticles, as determined by the Malvern instrument, averaged −14.45 mV ± 0.6mv (Fig. 2C). For stability assessment, the particle size and potential were monitored over 7 days at 4 °C storage. During this period, the particle size experienced a minor increase from 260.1 nm to 268.8 nm (Fig. 2D). Similarly, the potential shifted from an initial −14.45 mV to −13.63 mV by the end of the observation period (Fig. 2E).To further quantify the encapsulation efficiency of ^131^I-fulvestrant within the liposomes, a standard curve was established using fulvestrant as a reference. Through high-performance liquid chromatography, with the peak area plotted against concentration, the encapsulation efficiency of the 131I-fulvestrant in the prepared nanoparticles was determined to be 80.52 ± 4.68%, accompanied by a drug loading rate of 7.06 ± 0.54% (Fig. 2F). The results demonstrate that ^131^I-fulvestrant-ALA-PFP-FA-NPs have been successfully synthesized and exhibit stable properties.

### In vitro phase change capability and drug release behavior of 131I-fulvestrant-ALA-PFP-FA-NPs

**Fig. 3:**
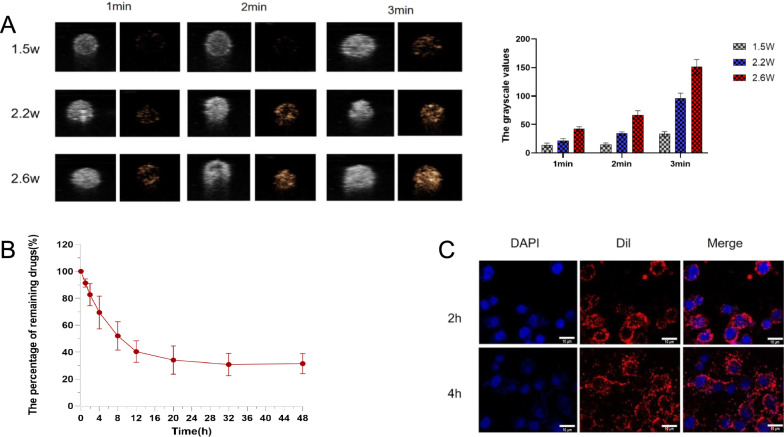
**In vitro characteristics of **^**131**^**I-fulvestrant-ALA-PFP-FA-NPs: A** Ultrasound imaging of nanoparticle morphology before and after LIFU irradiation. **B** DFY-type ultrasound image quantitative analysis diagnostic device for quantitative analysis of nanoparticle B-mode ultrasound and contrast-enhanced mode grayscale values. **C** Drug release kinetics of nanoparticles at different time points following LIFU irradiation. **D** Using confocal microscopy to observe in vitro targeting and transmembrane properties of DiI-labeled nanoparticles (Scale: 10 μm)(Values are means ± s.d., n = 3)

The nanoparticles were suspended in a 37 ℃ PBS buffer. Using ultrasonic imaging and microscopy, the phase changes and microbubble formations of these nanoparticles were examined under various parameters of LIFU. The results demonstrate that at 37 ℃ in the PBS buffer, the drug-loaded target liquid fluorocarbon nanoparticles undergo a distinct liquid–gas phase transition at an ultrasound power of 2.6 W/cm^2^ (Fig. 3A). Further analysis using the DFY-type ultrasonic image quantitative analysis diagnostic device revealed that as the LIFU irradiation intensity and duration elevate—particularly within the range of 1.5 W/cm^2^, 2.2 W/cm^2^, and 2.6 W/cm^2^—the ultrasonic grayscale values of the ^131^I-fulvestrant-ALA-PFP-FA-NPs correspondingly rise. The most notable escalation in both ultrasonic imaging and grayscale value occurs at 2.6 W/cm^2^ following a continuous 3-min irradiation (Fig. 3A). Assessing the in vitro drug release behavior of these nanoparticles, after being subjected to 2.6 W/cm^2^ LIFU for 3 min, a dialysis method was employed to chart their release pattern over 48 h. The drug release was rapid within the initial 16 h, reaching a peak at 30 h (Fig. 3B).

### In vitro tumor targeting and cytotoxicity study of ^131^I-fulvestrant-ALA-PFP-FA-NPs

**Fig. 4:**
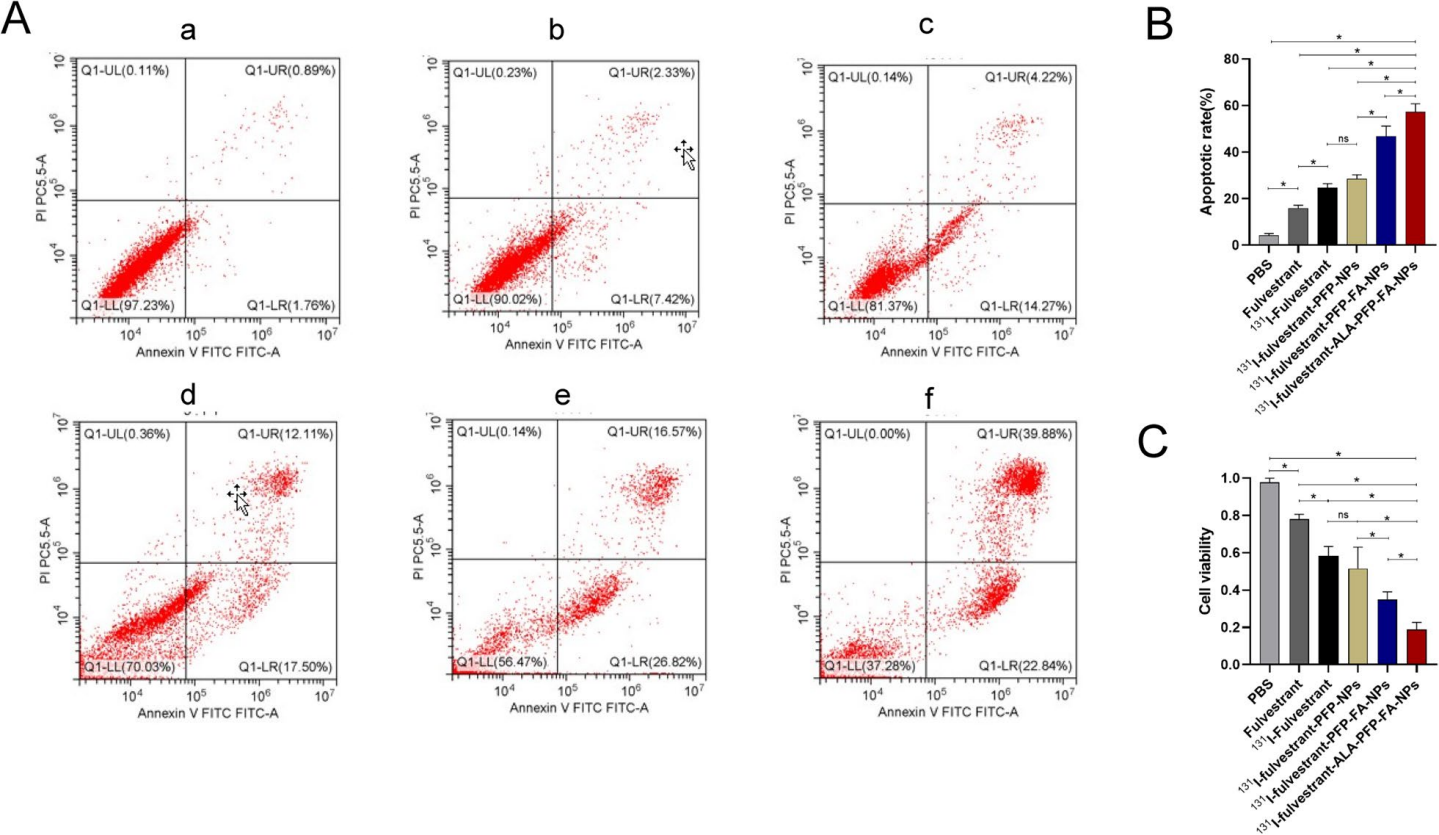
** Cytotoxicity study of **^**131**^**I-fulvestrant-ALA-PFP-FA-NPs: A** Flow cytometry analysis of apoptosis in different cell groups. **B** Semi-quantitative analysis of apoptosis in different cell groups. **C** Cell viability assessment of different cell groups using CCK-8 assay. a: PBS, b: Fulvestrant, c: 131I-fulvestrant, d: 131I-fulvestrant-PFP-NPs,e: 131I-fulvestrant-PFP-FA-NPs, f: 131I-fulvestrant-ALA-PFP-FA-NPs(Values are means ± s.d., n = 3)

The tumor-targeting capability of Dil-labeled ^131^I-fulvestrant-ALA-PFP-FA-NPs was examined by co-culturing them with MCF-7 cells, followed by evaluation through confocal microscopy and flow cytometry. Confocal imaging showcased a distinct Dil red fluorescent signal in the cytoplasm post-incubation, confirming that the nanoparticles exhibit efficient targeting of MCF-7 cells (Fig. 3C). Using the CCK-8 assay, we gauged the cytotoxic effects of the nanoparticles on ER( +) MCF-7 cells over varied durations. The observations pinpointed that the inhibitory impact of ^131^I-fulvestrant-ALA-PFP-FA-NPs on MCF-7 cells was most potent when aided by LIFU (Fig. 4C). Furthermore, flow cytometry was employed to assess the influence of the nanoparticles on the apoptosis of ER( +) MCF-7 cells over different intervals. The apoptosis rate, determined using the previously described methods and groupings, highlighted that the pro-apoptotic effect of ^131^I-fulvestrant-ALA-PFP-FA-NPs on MCF-7 cells was paramount (Fig. 4A, B). The results from this section demonstrate that ^131^I-fulvestrant-ALA-PFP-FA-NPs exhibit strong targeting and inhibitory capabilities against MCF-7 cells in vitro.

### Cerenkov radiation imaging and confocal detection of ALA to PpIX conversion

**Fig. 5:**
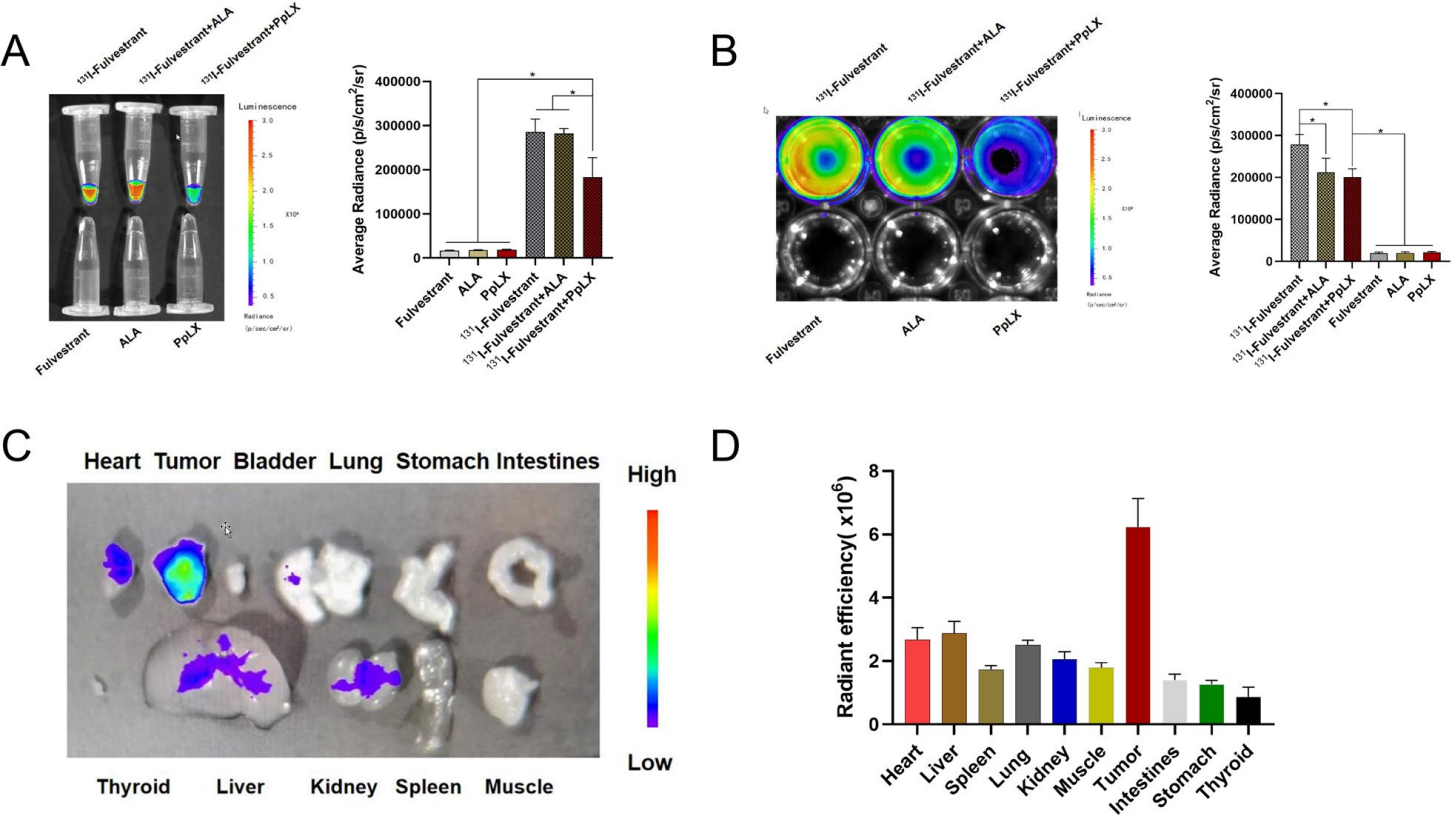
**Cerenkov radiation: A** Cherenkov radiation signal detection: Cherenkov radiation imaging and semi-quantitative analysis of different drug solutions. **B** Cherenkov radiation imaging and semi-quantitative analysis of different drug solutions added to MCF-7 cell culture dishes. **C** Fluorescence imaging of PpIX signal in excised tissues 24 h after nanoparticle injection. **D** Semi-quantitative analysis of PpIX signal in excised tissues

**Fig. 6:**
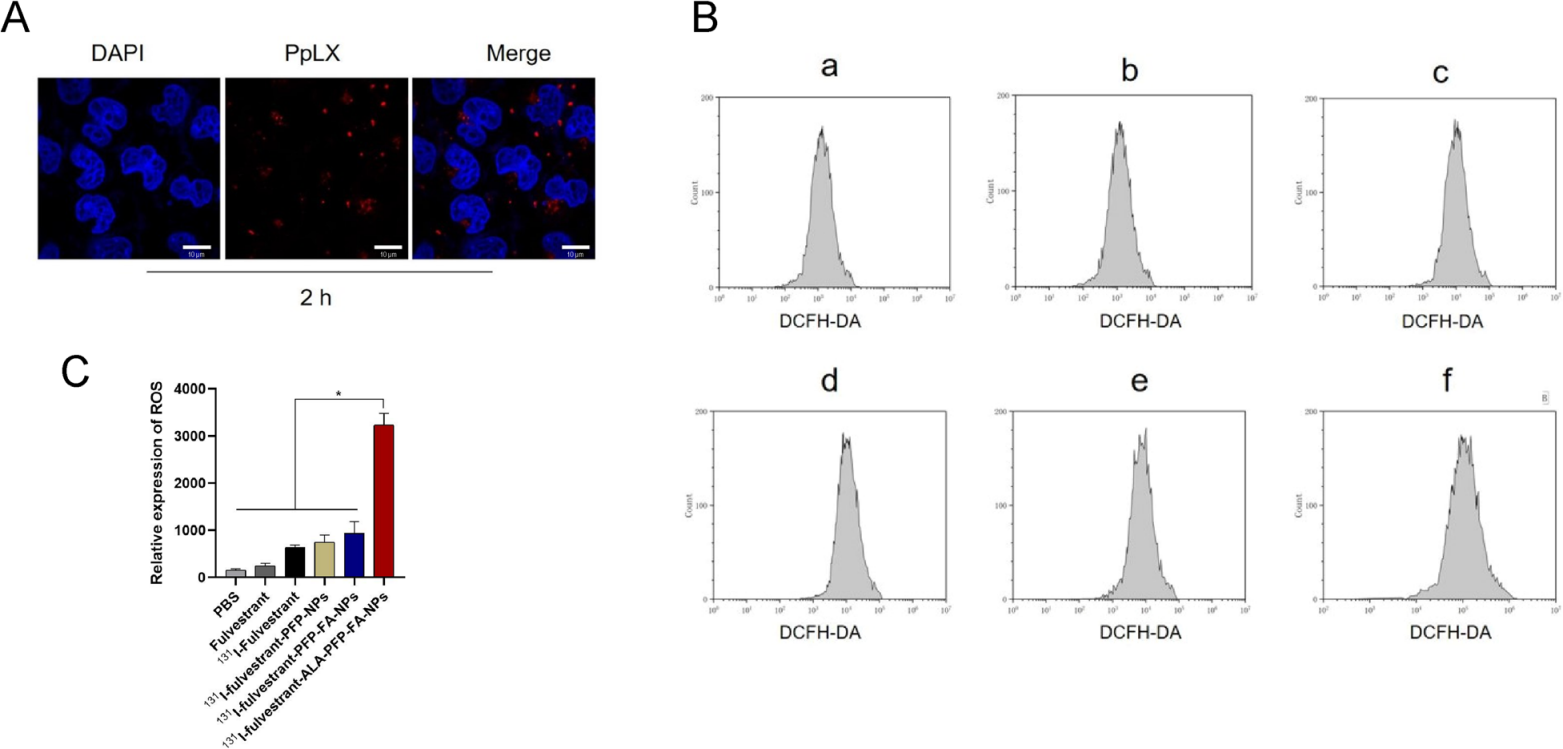
**Detection of ALA to PpIX conversion and ROS levels: A** Observation of red fluorescence of PpIX using confocal microscopy after 2 h of incubation with nanoparticles and MCF-7 cells. Scale = 50 μm. **B** flow cytometry analysis of ROS Levels in MCF-7 tumor cells after different treatment methods. **C** Quantitative analysis of ROS levels in MCF-7 tumor cells after different treatment methods using flow cytometry. (Values are means ± s.d., n = 5)

Utilizing an in vivo small animal imaging system (IVIS), we captured the signal of ^131^I without the use of excitation light to detect Cerenkov radiation (CR) and Cerenkov resonance energy transfer (CRET). Among the tested solutions, only those containing ^131^I, ^131^I + ALA, and ^131^I + PpIX exhibited noticeable fluorescence signals. Interestingly, the fluorescence intensity of the ^131^I + PpIX solution was diminished compared to the ^131^I + ALA solution (p < 0.05) (Fig. 5A). This phenomenon can be attributed to PpIX absorbing the photons from CR, which triggers CR-PDT, subsequently resulting in fewer photons being captured by the IVIS system. For further investigation, we introduced solutions of ^131^I, ^131^I + ALA, and ^131^I + PpIX into a 12-well plate containing MCF-7 cells. Observationally, the ^131^I + ALA group's fluorescence signal was found to be fainter than the ^131^I + PpIX group, suggesting that ALA in tumor cells can be converted to PpIX in vitro, thereby activating PDT. Laser confocal imaging further confirmed the conversion of intracellular ALA to PpIX (Fig. 5B). Post a 2-h incubation of ^131^I-fulvestrant-ALA-PFP-FA-NPs with MCF-7 cells, the distinctive red fluorescence signal of PpIX was detected in the MCF-7 cells (Fig. 6A). For successful CR-PDT, the Cerenkov radiation wavelength needs to align with the photosensitizer excitation wavelength. The conducted experiments affirmed that the spectrum of PpIX harmonizes well with Cerenkov light, endorsing it as an apt photosensitizer for CR-PDT. Exploring the in vivo conversion of ALA to PpIX, it was observed that 24 h post the injection of ^131^I-fulvestrant-ALA-PFP-FA-NPs, the fluorescence signals of PpIX were predominantly in tumors, with slight accumulation observed in the liver and heart (Fig. 5C). Lastly, ROS levels across different cell groups were examined. MCF-7 cells treated with ^131^I-fulvestrant-ALA-PFP-FA-NPs displayed a pronounced increase in ROS levels, ranging between 2 and 7 times compared to other groups. This reinforces the notion that ^131^I-fulvestrant-ALA-PFP-FA-NPs potentiate cytotoxicity through the induction of ROS production (Fig. 6B).

### In vivo distribution and tumor-targeting capability of ^131^I-fulvestrant-ALA-PFP-FA-NPs

**Fig. 7:**
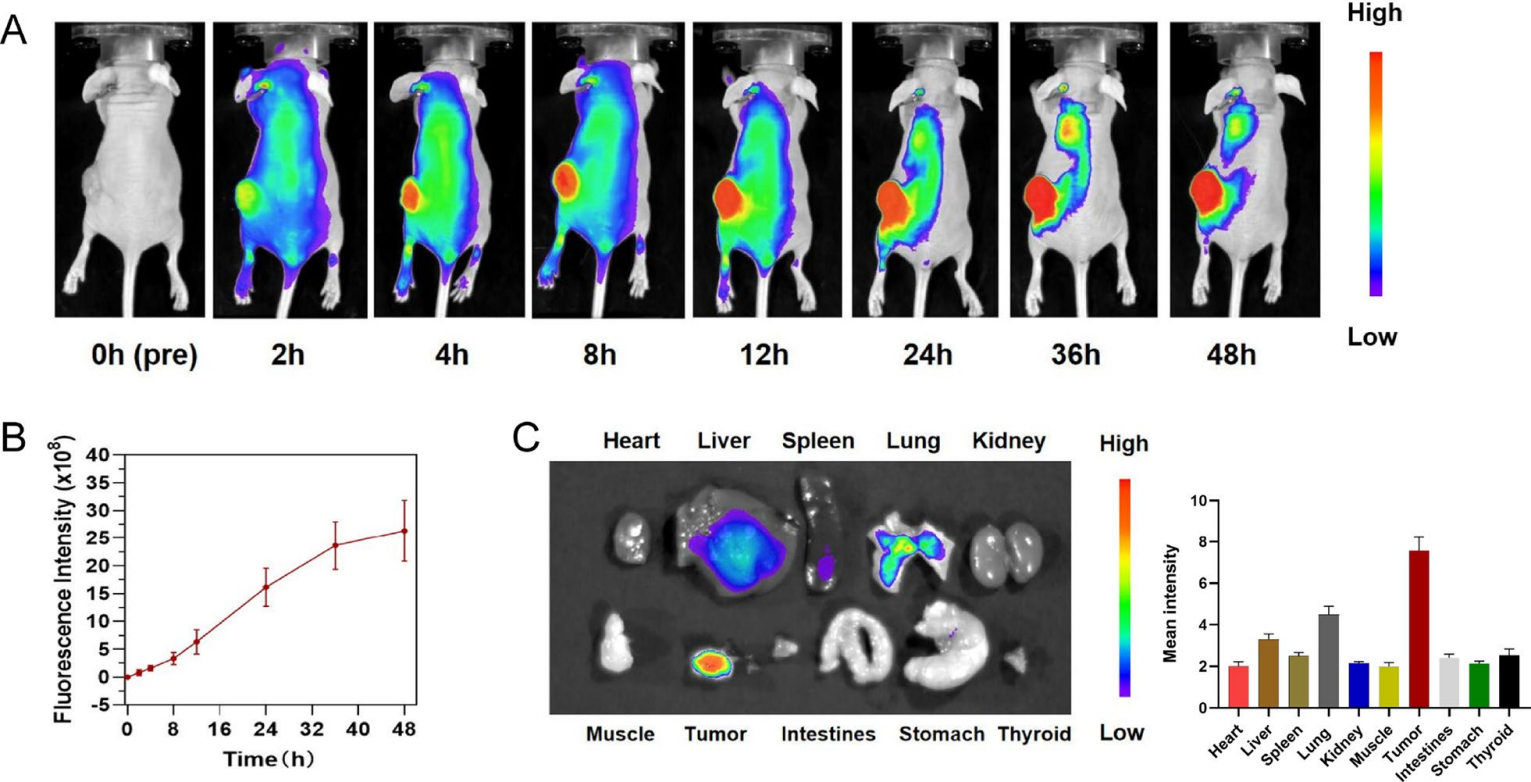
**In vivo and in vitro imaging of **^**131**^**I-fulvestrant-ALA-PFP-FA-NPs: A** In vivo fluorescence imaging of tumors in mice bearing tumors at different time points following intravenous injection of ^131^I-fulvestrant-ALA-PFP-NPs. **B** Changes in fluorescence signal intensity at corresponding time points within the tumor region. **C** Fluorescence imaging of excised major organs 24 h after intravenous injection of.^131^I-fulvestrant-ALA-PFP-NPs in mice bearing tumors. **D** Semi-quantitative analysis of average fluorescence intensity in various organs and tumors (Values are Mean ± S.D., n = 5)

**Fig. 8:**
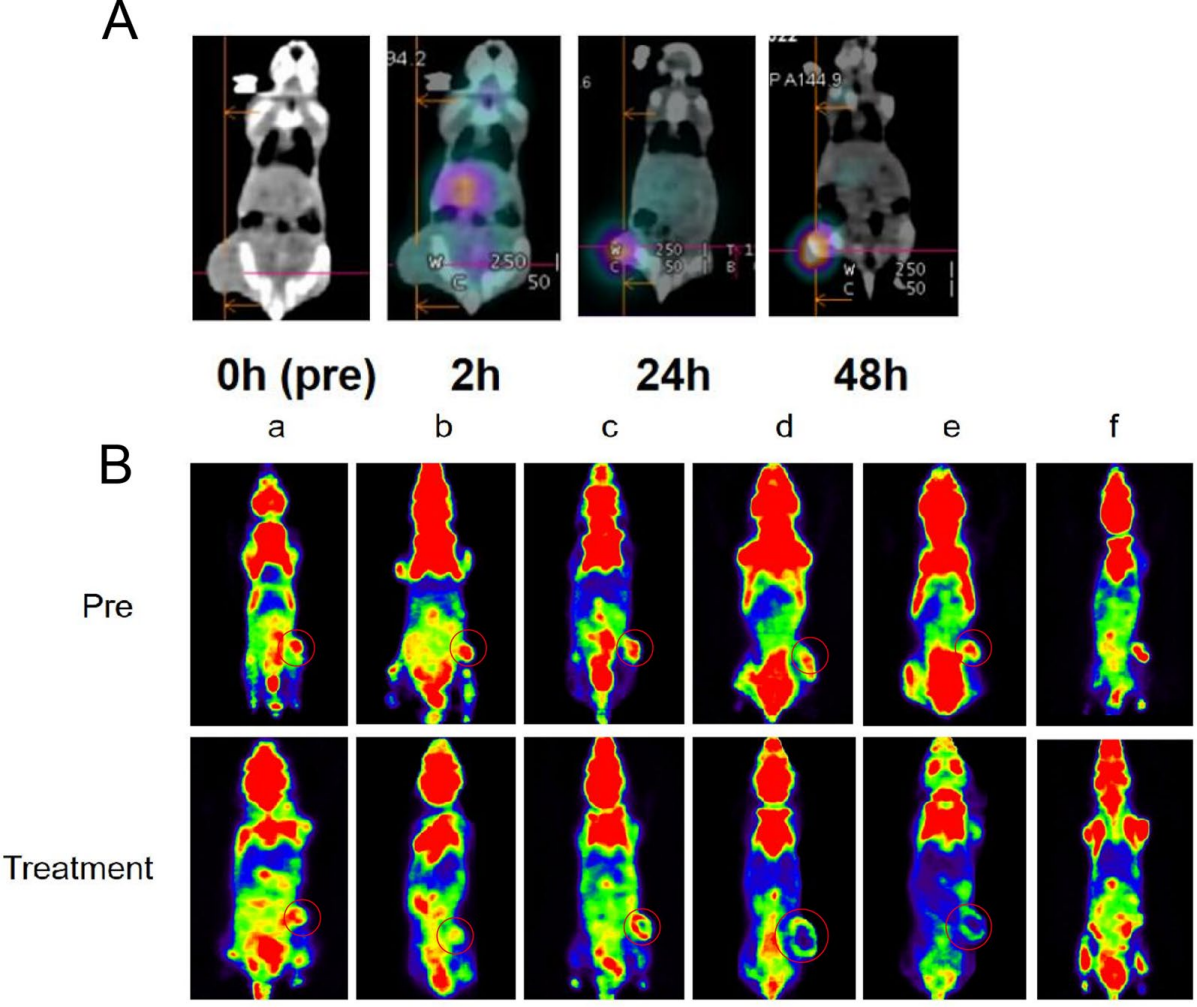
**In vivo tumor-targeting capability and antitumor efficacy of **^**131**^**I-fulvestrant-ALA-PFP-FA-NPs: A** Detection and biodistribution of nanoparticle targeting to tumors at different time points after intravenous injection of ^125^I-fulvestrant-ALA-PFP-NPs Using SPECT/CT. **B** PET/CT analysis of FDG uptake in tumor tissues before and after various treatment groups. (Values are Mean ± S.D., n = 5)

To ascertain the in vivo distribution of nanoparticles, ^131^I-fulvestrant-ALA-PFP-FA-NPs were intravenously administered into MCF-7 tumor-bearing mice. Intriguingly, the tumor's fluorescence signal amplified progressively over time, peaking at the 48-h mark. This enhancement pattern hints at a time-dependent accumulation of DIR-labeled ^131^I-fulvestrant-ALA-PFP-FA-NPs within the tumor (Fig. 7A, B). By 48 h post-injection, the Tumor-to-Muscle (T/M) ratio reached its zenith at 1.62 ± 0.11. Post euthanization at the 48-h juncture, the dissected organs were subjected to fluorescence imaging, reaffirming the pronounced aggregation of ^131^I-fulvestrant-ALA-PFP-FA-NPs in the tumor vicinity. Radiological activity assessments further validated this accumulation (Fig. 7C). It's noteworthy to mention the prominent fluorescence detected in the liver, potentially indicative of hepatic clearance mechanisms processing the ^131^I-fulvestrant-ALA-PFP-FA-NPs nanoparticles. To bolster the evidence of tumor-centric accumulation of ^131^I-fulvestrant-ALA-PFP-FA-NPs, ^131^I was substituted with ^125^I for a more in-depth SPECT/CT imaging analysis. Post the 72-h injection window, images unveiled that from the 24-h marker, the signal intensity within the tumor surpassed that of the adjoining tissues (Fig. 8A). Conclusively, both imaging and biodistribution data firmly establish that ^131^I-fulvestrant-ALA-PFP-FA-NPs predominantly congregate within the tumor region.

### In vivo antitumor efficacy and histological examination of ^131^I-fulvestrant-ALA-PFP-FA-NPs

**Fig. 9:**
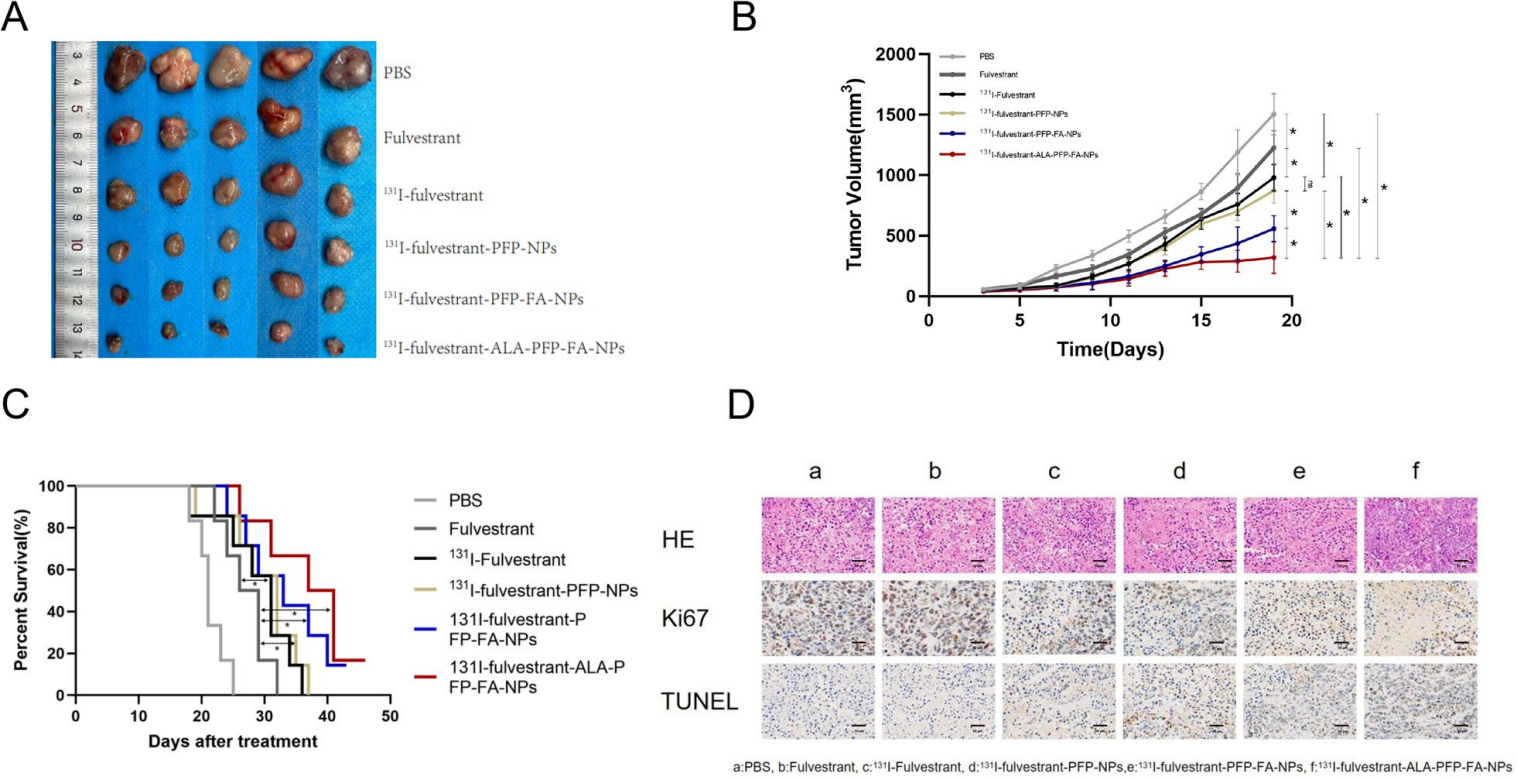
**In vivo antitumor efects of **^**131**^**I-fulvestrant-ALA-PFP-FA-NPs: A** Evaluation of in vivo antitumor effects in different drug treatment groups: changes in tumor size following different treatments. **B** Growth curves of tumors in different drug treatment groups following various treatments. **C** Survival curves of tumor-bearing mice in different drug treatment groups following various treatments. **D** Histological evaluation of tumor tissues in different drug treatment groups: he staining, Ki67 staining, and TUNEL staining. a: PBS, b: Fulvestrant, c: 131I-fulvestrant, d: 131I-fulvestrant-PFP-NPs,e: 131I-fulvestrant-PFP-FA-NPs, f: 131I-fulvestrant-ALA-PFP-FA-NPs(Scale bar represents 50 μm for all panels, Values are Mean ± S.D., n = 5)

We evaluated the in vivo antitumor effect of ^131^I-fulvestrant-ALA-PFP-FA-NPs, especially considering its impressive tumor penetration capability enhanced by LIFU, using an MCF-7 tumor-bearing nude mouse model. The animals were divided into six groups, each receiving varied treatments (details of grouping are in the methods section). Observations highlighted that the control group, treated with PBS, experienced the most significant tumor growth, with tumor volume amplifying by 10 times. In contrast, the fulvestrant group saw an eightfold increase in tumor volume. Notably, the ^131^I-fulvestrant group demonstrated improved tumor suppression, showing a sixfold tumor volume increase. The most significant tumor suppression was observed in the ^131^I-fulvestrant-ALA-PFP-FA-NPs group, where tumor volume rose only 1.8 times (Fig. 9A, B). This heightened therapeutic effect is credited to the targeted nanomedicine approach using ^131^I-fulvestrant-ALA-PFP-FA-NPs, facilitated by folic acid targeting and the intrinsic EPR effect. Under the aid of LIFU irradiation, PFP within the ^131^I-fulvestrant-ALA-PFP-FA-NPs undergoes a liquid–gas phase transition. This causes the release of ^131^I-fulvestrant and ALA, enhancing the permeability of tumor cells and subsequently boosting drug uptake. Survival curve analysis further substantiates the potency of this treatment, confirming that it can significantly extend the mice's survival duration (Fig. 9C). To shed light on the underlying effects, we executed ^18^F-FDG PET/CT imaging on the 21st day post-treatment across all groups with MCF-7 tumors. A visual assessment revealed a noticeably reduced ^18^F-FDG uptake in the ^131^I-fulvestrant-ALA-PFP-FA-NPs group when juxtaposed with the control. This suggests a decline in the cellular metabolic rate of treated tumors, resulting in tumor regression (Fig. 8B). Further histological analyses using Hematoxylin and Eosin (H&E) stained tumor sections provided insights into the therapeutic impacts of various treatments. The TUNEL and PCNA immunohistochemical tests unveiled that the group receiving ^131^I-fulvestrant-ALA-PFP-FA-NPs combined with LIFU exposure presented the highest apoptosis index (*p < 0.05) and the lowest proliferation index (*p < 0.05) (Fig. 9D).

### Examination of the biosafety of ^131^I-fulvestrant-ALA-PFP-FA-NPs

**Fig. 10:**
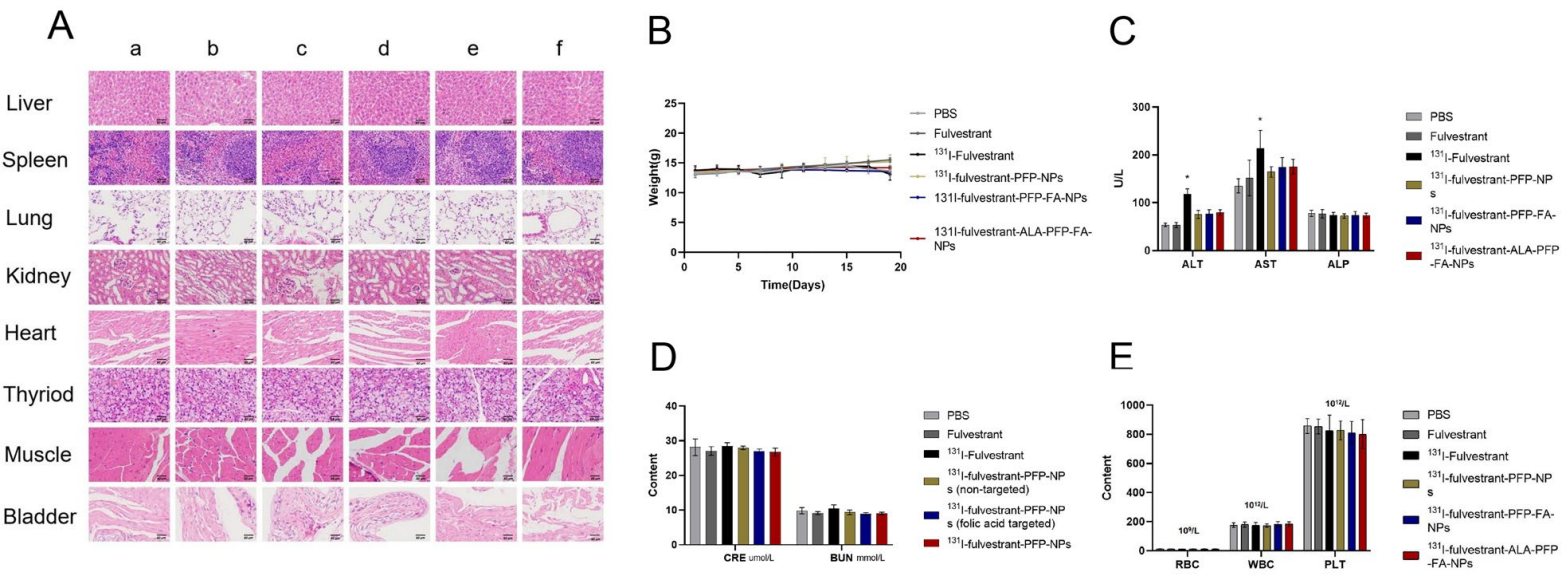
**In vivo toxicity test: A** Safety assessment of drug: HE staining of major organs. **B** Changes in body weight of experimental mice in each treatment group during administration. **C** Impact of different drug treatments on liver function biomarkers in mice: alanine aminotransferase (ALT), aspartate aminotransferase (AST), and alkaline phosphatase (ALP). **D** Impact of different drug treatments on renal function biomarkers in mice: blood urea nitrogen (BUN) and creatinine (CRE). **E** Impact of different drug treatments on hematological parameters in mice: white blood cells (WBC), red blood cells (RBC), platelets (PLT).a:PBS, b:Fulvestrant, c:131I-Fulvestrant, d:131I-fulvestrant-PFP-NPs,e:131I-fulvestrant-PFP-FA-NPs, f:131I-fulvestrant-ALA-PFP-FA-NPs(Scale bar represents 50 μm for all panels, Values are Mean ± S.D., n = 5)

To ensure the in vivo therapeutic safety of ^131^I-fulvestrant-ALA-PFP-FA-NPs in terms of tumor cytotoxicity and treatment response, histological examinations were conducted on vital organs, including the heart, liver, spleen, lungs, kidneys, and thyroid. Following the various treatments, these organs were subjected to H&E staining. The results displayed no remarkable histopathological alterations, confirming the tissue compatibility of 131I-fulvestrant-ALA-PFP-FA-NPs (Fig. 10A).To delve deeper into the potential in vivo toxicity, we studied the impact of ^131^I-fulvestrant-ALA-PFP-FA-NPs over both short-term and extended durations. Observations such as minimal fluctuations in body weight (Fig. 10B), consistent blood biochemical indicators (Fig. 10C–E), and the absence of notable changes in the H&E staining of major organs (Fig. 10A) suggest an absence of discernible toxicity in the mice. These findings, taken collectively, highlight the robust therapeutic biosafety of ^131^I-fulvestrant-ALA-PFP-FA-NPs when applied to breast cancer treatments.

## Discussion

Endocrine therapy and radiotherapy play pivotal roles in the comprehensive treatment of malignant breast tumors. With the recent rise in breast-conserving surgeries, radiotherapy's significance in breast cancer treatment has intensified [34]. Traditional methods, which rely on external irradiation of the tumor area using rays from linear accelerators, unfortunately, present a risk of severe radiative side effects [35]. Consequently, the scientific community has been in pursuit of safer and more precise radiotherapy modalities. In this context, using radionuclides for targeted and controllable radiotherapy has emerged as a preferred option [36]. Although innovations such as interstitial brachytherapy, intratumoral injection of radioactive isotopes, and radioactive iodine oil embolization of tumor blood vessels have been introduced, they share the inherent limitation of radiotherapy being primarily a localized treatment [37]. However, with advancements in monoclonal antibodies and nanotechnology, targeted radionuclide therapy (TRNT) has shown great promise. In TRNT, therapeutic radionuclides bind to specific carriers, concentrating in tumor cells. This not only ensures targeted radiation to eradicate tumor cells but also safeguards surrounding healthy tissues [38]. Notably, ^131^I, which primarily (99%) utilizes β rays for its therapeutic radiative effect, has an average range of just 0.48 mm. This ensures minimal damage to adjacent tissues, highlighting its potential for reduced radiation-induced side effects [39]. Researchers have effectively harnessed the properties of ^131^I by coupling it with tumor markers, monoclonal antibodies, and cell-specific drugs. This has led to extensive exploration of ^131^I carrier markers for malignant tumor treatment [40]. For instance, studies have showcased the safe and significant effectiveness of ^131^I-anti CD45, ^131^I-anti CD22, and I-Rituximab in treating non-Hodgkin's lymphoma [41–43]. The ^131^I-B lymphoma Fab antibody has proven instrumental for early ECT tumor imaging and precise tumor localization, demonstrating its potential to effectively inhibit tumor growth [44]. In the realm of breast cancer research, there have been advancements in the utilization of radioactive iodine markers. Notably, experiments by Lin Jing and colleagues have revealed that ^131^I-Herceptin exhibits in vitro cytotoxic effects on Her-2 positive breast cancer cell lines [45]. Similarly, research by Xu Weiyun et al. has highlighted the considerable inhibitory impact of ^131^I-epidermal growth factor (^131^I-EGF) on tumor cells in nude mouse breast cancer xenografts [46]. Endocrine therapy stands as a primary treatment for hormone-dependent breast cancer. It often centers around either inhibiting estrogen production or counteracting estrogen's effects. Fulvestrant, a pioneering steroidal estrogen receptor antagonist, has been approved for postmenopausal women with estrogen receptor-positive advanced metastatic breast cancer, particularly after an initial endocrine therapy [47]. Its mode of action involves competitive binding with estrogen to the ER, inhibiting post-receptor effects. Impressively, it also downregulates the numbers of estrogen and progesterone receptors at the cellular level. Its endocrine anticancer efficacy surpasses traditional anti-estrogen drugs, such as tamoxifen, given its estrogen receptor binding affinity is a staggering 300 times greater than tamoxifen [48]. Nevertheless, while fulvestrant's primary role is to stifle tumor cell growth, it doesn't directly eradicate tumor cells.Consequently, when cancer cells develop resistance to fulvestrant, challenges related to recurrence and metastasis persist [48]. Moreover, Fulvestrant's limited solubility poses challenges for its delivery and therapeutic effectiveness. Achieving stable plasma concentrations of 24–28 ng/mL requires intramuscular injections [49], which can lead to discomfort at the injection site, including sciatic neuralgia and peripheral neuropathy [50], adversely affecting the patient's quality of life. The standard 200 mg/5 mL concentration of commercial fulvestrant necessitates intramuscular injection (IM), yet clinical data suggest that even the maximum tolerable dose of 500 mg per treatment is insufficient for completely blocking the ERα receptor [51]. Compounding these issues is Fulvestrant's low oral bioavailability, which further constrains its clinical utility and underscores the need for improved formulations and delivery methods to enhance its efficacy in combating breast cancer.

Building on the insights from previous research, we innovatively labeled the fulvestrant molecule with ^131^I, transforming it into a radioactive ^131^I carrier. Given the fulvestrant's specificity in binding to estrogen receptors, the radioactive iodinated fulvestrant is strategically positioned to target estrogen receptor-positive breast cancer cells. This culminates in a potent synergy between radiation therapy and endocrine therapy. Our preliminary trials revealed that ^131^I-fulvestrant exerts a significantly enhanced inhibitory action on breast cancer cells compared to unaltered fulvestrant.

However, a recurring challenge with most ^131^I-conjugated drugs, ^131^I-fulvestrant included, is their suboptimal targeting. Estrogen receptors, while abundant in tumor cells, are also ubiquitously expressed across various human organs. As a result, organs expressing estrogen receptors may inadvertently be harmed by the radioactive iodine, manifesting potential side effects [52]. Our solution to this targeting conundrum leverages the targeted drug delivery system of fluorocarbon nanoparticles. We devised a composite by integrating ^131^I-fulvestrant with fluorocarbon phase-change nanoparticles conjugated with folic acid. This resultant radioactive ^131^I-fulvestrant-fluorocarbon nanoparticle composite is adept at homing in on tumor sites, courtesy of its enhanced permeability and retention capabilities [53]. Moreover, its affinity for breast cancer cells is heightened due to the presence of folic acid receptors on these cells. The inclusion of perfluoropentane (PFP) further amplifies the composite's efficacy; PFP induces a liquid–gas phase transition that creates a "burst" effect, elevating intracellular drug concentrations [54]. In comparative studies, the ^131^I-fulvestrant-PFP-FA-NPs targeted nanoparticles showcased superior cytotoxicity against tumor cells relative to their non-targeted counterparts.

Photodynamic therapy (PDT) is a modulated therapeutic technique that necessitates external stimulation. It operates by exposing photosensitizers (PSs) to a specific light wavelength, facilitating the generation of reactive oxygen species (ROS) that exterminate cancer cells [55]. However, the penetration depth of light pivotal for PDT is confined, rendering it inefficient for activating photochemical reactions in deeply embedded tumors [56]. This limitation severely restricts its efficacy for treating tumors situated deeper within the body. In clinical settings, surgical excision remains the primary approach for managing primary tumor growths, with pharmacological interventions primarily targeting post-surgical residual or metastasized tumor cells [57]. The indispensable requirement for exogenous light in PDT curtails its broader clinical applicability [58]. Yet, a novel avenue arises with PDT stimulated by Cerenkov radiation (CR) emanating from the radioactive decay process, termed CR-PDT. Cerenkov radiation, an illuminating event associated with radioactive nuclide decay, enables CR in proximity to radioactive elements to activate PSs, leading to the generation of cytotoxic ROS. This innovative strategy emerges as a potential game-changer for addressing deeply entrenched or metastatic tumors [59, 60]. To further refine this technique, and to prevent premature photosensitizer activation by Cerenkov radiation during drug synthesis, we innovated a molecular switch capitalizing on the tumor's mitochondrial abundance. By conjugating the photosensitizer precursor ALA, a natural forerunner to PpIX, onto nanoparticles, we established a sequential activation process. Once these nanoparticles arrive at the tumor vicinity, the tumor's mitochondria facilitate the conversion of ALA to PpIX. Subsequently, PpIX is triggered by the CR derived from 131I decay, producing ROS that effectively eliminates tumor cells [61]. It's noteworthy that in typical tissues, the mitochondrial concentration is sparser, inhibiting the ALA to PpIX transition, ensuring that side effects remain minimal.

In our comprehensive experimentation, the nanoparticles showcased remarkable structural robustness. They remained consistent in size, with negligible enlargement even after a 7-day duration at ambient temperature. In a PBS environment, the radiochemical attributes of the ^131^I-fulvestrant-ALA-PFP-FA-NPs liposomes remained unaltered, underscoring their stability. In plasma settings, upon the application of low-power focused ultrasound at an intensity of 2.6 W/cm^2^, these drug-laden fluorocarbon nanoparticles underwent a pronounced transition from liquid to gas phase. Through both in vitro and in vivo investigative avenues, it was evident that the ^131^I-fulvestrant-ALA-PFP-FA-NPs exhibited commendable targeting capabilities. They also displayed potent cytotoxic properties against hormone receptor-affirmative MCF-7 cells. An ancillary revelation was that these nanoparticles facilitated the release of ALA within tumor matrices. This enabled its seamless conversion to PpIX inside the tumor environment, leading to ROS generation upon the influence of CR. This process culminated in an effective photodynamic response. Subsequent analysis across various tissue types indicated that these nanoparticles possess favorable biocompatibility. Importantly, they were devoid of any significant adverse manifestations.

In our investigation, we integrated fulvestrant with the radioactive isotope ^131^I and utilized fluorocarbon nanoparticles conjugated with folic acid to target breast cancer delivery effectively. This method significantly enhanced the antitumor effect. Our nanocomplex uses the folic acid receptor as its primary target, directing drug-loaded nanoparticles precisely to breast cancer sites. Employing LIFU irradiation, it induces a phase transition in the tumor region, leading to the destruction of cancer cells and enhancing cellular permeability. This process facilitates the targeted release of ^131^I-fulvestrant. Additionally, the unique binding capability of fulvestrant to estrogen receptors enables a second precise targeting of breast cancer cells. This approach not only markedly improves the antitumor effect but also addresses the low bioavailability of fulvestrant and the insufficient targeting of ^131^I in breast tumors, showcasing the potential of a novel treatment strategy for breast cancer.

Our nanomedicine demonstrates clear advantages in particle size, biocompatibility, safety, and tumor targeting over existing strategies widely explored in the literature, such as FA-liposome nanomedicines for FR targeting and nanotherapeutic approaches combining fulvestrant with other drugs. Compared to the Liposomes Containing ^131^I as Cargo developed by Kolašinac R et al. for targeted therapy of breast cancer cells (particle size: 456 nm), our nanoparticles are smaller (260.1 nm), showing higher biocompatibility [62]. They exhibit similar in vivo antitumor efficacy and biosafety to the folic acid-targeted nanostructures studied by Kefayat A et al. and comparable tumor-to-muscle ratios (T/M ratios) to the 131I-related tumor-targeted nanoparticles (131I-EM@ALA) prepared by Qian R et al., all indicating good tumor targeting [18, 32].

In our nanoparticle platform, we co-encapsulated fulvestrant and ^131^I in an optimal ratio for precise tumor delivery. Compared to traditional intramuscular injections with low bioavailability and associated pain and side effects, our ^131^I-fulvestrant-ALA-PFP-FA-NPs enable a burst release of ^131^I-fulvestrant in the tumor microenvironment, potentially increasing or maintaining therapeutic effects while reducing side effects. This provides a more comfortable treatment experience for patients. Our nanoparticles are structurally stable, exhibiting significant antitumor activity without noticeable adverse effects. Using ALA-induced photosensitizer PpIX via CR stimulation, our strategy effectively inhibits tumor growth while minimizing side effects on normal organs, especially the liver and blood cells. We found that the conversion of ALA to PpIX occurs primarily at tumor sites, reducing transformation in normal tissues. This offers a new pathway for photodynamic therapy independent of external light sources, significantly reducing side effects.

In conclusion, our research not only advances targeted treatment efficacy but also opens new avenues for nanotherapeutic strategies in breast cancer. Our findings promise a significant improvement in breast cancer treatment efficiency and patient comfort.

## Conclusion

We have successfully synthesized the nanoparticle, ^131^I-fulvestrant-ALA-PFP-FA-NPs, designed for the efficient delivery of iodinated fulvestrant. By exploiting folic acid receptors for primary targeting and leveraging the EPR effect, this design precisely directs the drug-laden nanoparticles toward breast cancer lesions. LIFU irradiation induces a phase transition within the tumor, resulting in cellular rupture and tumor cell death. This enhances cellular permeability and ensures the targeted release of iodinated fulvestrant. Capitalizing on fulvestrant's specificity to bind estrogen receptors, this system offers a secondary precision targeting mechanism. Notably, ^131^I functions both as a radiotherapeutic agent and, through its Cerenkov radiation (CR), serves as an internal light source. This paves the way for photodynamic effects driven by PpIX, introducing an innovative breast cancer treatment that combines targeted radiation, endocrine regulation, and photodynamic therapy, heralding a new era in breast cancer treatment.

## Data Availability

All datasets are available upon reasonable request.

## Abbreviations

FA: Folic acid
PFP: Perfluoropentane
DPPC: Dipalmitoylphosphatidylcholine
DSPE-PEG2000: 1,2-Distearoyl-sn-glycero-3-phosphoethanolamine-N-[methoxy(poly(ethylene glycol))- 2000]
DiI: 1,1'-Dioctadecyl-3,3,3',3'-Tetramethylindocarbocyanine perchlorate
DAPI: 4',6-Diamidino-2-phenylindole
TRNT: Targeted radionuclide therapy
LIFU: Low-intensity focused ultrasound
PDT: Photodynamic therapy
CR: Cerenkov radiation
CR-PDT: The Cerenkov radiation-induced PDT
ALA: 5-Aminolevulinic acid
ROS: Reactive oxygen species
PpIX: Protoporphyrin IX
TEM: Transmission electron microscope
SPECT/CT: Single-photon emission computed tomography/computed tomography
AST: Aspartate aminotransferase
ALT: Alanine aminotransferase
BUN: Blood urea nitrogen
CRE: Creatinine
ALP: Alkaline phosphatase
WBC: White blood cells
RBC: Red blood cells
PLT: Platelets
H&E staining: Hematoxylin and eosin staining
TUNEL staining: Terminal deoxynucleotidyl transferase dUTP Nick End Labeling staining
SD: Standard deviation
